# The associations between public stigma and support for others’ help-seeking for alcohol use disorder: a cross sectional study in the general Danish population

**DOI:** 10.1186/s13722-023-00400-2

**Published:** 2023-08-04

**Authors:** Sara Wallhed Finn, Anna Mejldal, Anette Søgaard Nielsen

**Affiliations:** 1 Unit of Clinical Alcohol Research, Institute of Clinical Research, University of Southern Denmark, J.B. Winsløws Vej 20, entrance. 220 B, 5000 Odense, Denmark; 2 Department of Global Public Health, Karolinska Institutet, Stockholm, Sweden; 3 Psychiatric Hospital, University Function, Region of Southern Denmark, Odense, Denmark

**Keywords:** Alcohol-related disorders, Health services research, Population, Europe

## Abstract

**Background:**

A minority of all individuals with alcohol use disorders (AUD) seek treatment, where stigma is one prominent barrier. Social support is important to facilitate health and increase treatment-seeking. Whether there is an association between stigma and attitudes towards others’ help-seeking for AUD is unknown. The aim of this study was to investigate the associations between stigma and support towards others’ help-seeking for AUD, also to explore possible gender differences.

**Method:**

Cross-sectional study, n = 2895, including Danish adults aged 30–65 in the general population. Year 2020, an online questionnaire was administrated, which covered demographics, attitudes towards others’ help-seeking for AUD, and stigma measured with the Difference, Disdain & Blame Scales. Analyses were performed with Restricted Cubic Spline models, and odds ratios were calculated.

**Results:**

Lower level of stigma was associated with a higher probability for endorsing an “active support strategy”. Level of stigma was not associated with “not knowing what to say or do” or “sharing my concern with others”. There were few gender differences: among men, higher level of stigma was associated with a higher probability of “avoidance”. Among women, lower level of stigma was associated with a lower probability of “avoidance”.

**Conclusion:**

There is a clear association between stigma and attitudes towards supporting others’ help-seeking for AUD. The results highlight the need to reduce stigma and promote engagement towards others’ treatment-seeking.

**Supplementary Information:**

The online version contains supplementary material available at  10.1186/s13722-023-00400-2.

## Background

Alcohol use disorder (AUD) is among the most highly stigmatized medical conditions in the Western world [1, 2]. Public stigma is defined as negative perceptions and stereotypes towards a specific group by the majority [3]. One key aspect of stigma is the perception of differentness, i.e., the affected group is viewed as being different from the majority [4, 5]. This perception of differentness is argued to be a stereotype shared between different stigmatized conditions. Moreover, for stigma to be present, rather than a neutral statement of difference, the perception of differentness needs to lead to disdain [6, 7]. A person belonging to the stigmatized group may internalise the public stigma, a process called self-stigma [8]. In the present paper, when stigma is discussed, it is public stigma, i.e., perceptions shared in the general population, that is referred to.

Some manifestations of stigma, as discrimination, lead to serious consequences for the affected group with diminished opportunities for achieving personal goals such as education, work, and housing [9]. Both the World Health Organization (WHO) and the Substance Abuse and Mental Health Services Administration (SAMHSA) in the United States have emphasized the need for more attention to stigma associated with substance use disorders (SUD), in particular how stigma affects individuals suffering from AUD [10, 11].

AUD has, compared to other psychiatric disorders, one of the largest gaps between the number of affected individuals and the number of individuals seeking treatment [12, 13]. This is also the case in Denmark, despite the fact that treatment services for AUD are free of charge and readily available [14]. It is important to reach a larger proportion of individuals with AUD with treatment in order to reduce alcohol-related harms, but barriers for treatment-seeking are high. It is well established that stigma associated with AUD is perceived as one of the most prominent barriers to treatment-seeking [15–17]. Stigma is also a barrier on the health care provider side, and is reported to refrain health care professionals to ask about patient’s alcohol use [18]. This can also perpetuate the treatment gap, as patients are neither being made aware of the potential risk cause by the alcohol intake, nor of the possibilities for help. Low problem recognition is a commonly reported barrier to treatment seeking [19].

Social support is a facilitator of good mental and physical health, particularly in the presence of stressors [20, 21]. Support from others can be a means to address the stressors and may also enhance self-efficacy [22]. Among individuals in treatment for SUD, higher perceived social support from family, friends and significant others is associated with lower level of internalized stigma, suggesting that social support also can be protective against self-stigma processes [23]. Moreover, social support for help-seeking behaviors has been found important for those suffering from SUD to take the step to seek help [24–26]. Therefore, one way to narrow the treatment gap for AUD might be to enhance social support for help-seeking. However, a cross-study from Taiwan shows that among individuals in treatment for SUD, perceiving a higher level public stigma is associated with lower perceived social support from family, friends and significant others [27]. This highlights the need to improve our understanding of the associations between public stigma and social support. Thus far, there has been little attention to the relationship between stigma and the offering of social support. Whether there is an association between stigma and attitudes towards encouraging or supporting others’ help-seeking for AUD in the general population is yet unknown.

## Method

### Aim

The overall aim of this study is to investigate if there are associations between perceived public stigma and attitudes towards supporting individuals, suffering from AUD, in seeking help for their alcohol problems. Moreover, possible gender differences will be explored, as previous studies suggest that women endorse lower level of stigma compared to men [28].

The analyses will take demographic factors, age, education, and previously having alcohol problems into consideration.

### Study design

Cross-sectional study.

### Participants

The participants were recruited by a market research company with access to a panel consisting of adults from all regions in Denmark, representing the general population. Between June and October 2020, a group of adults aged 30–65 years was asked to participate in an online questionnaire. The topic of the survey was not known to the participants beforehand, and took circa 12 to 16 min to complete. The proportion of participants who dropped out before completing the survey was slightly higher compared to surveys from the same market research company of similar length on other topics: 8.5% compared to normally 5–6%. In total, 2895 individuals participated.

### Outcome

The outcome measures were attitudes towards others’ help seeking for AUD. Please see “Measurements” section for a full description of attitudes assessed.

### Measurements

The questionnaire covered demographic data on sex, age category, education and having children. Experience of having alcohol problems was measured with the item: “Have you previously had alcohol problems?”, where the participants could answer “yes” or “no”.

Public stigma was measured with the Difference, Disdain & Blame Scales for Public Stigma, a questionnaire where the participant rate nine items on a scale from one (not at all) to nine (very much). The Difference and Disdain scale has been used in previous studies [6, 29, 30], and show good internal consistency [6]. For the purpose of the present study, the items were rephrased from “mental illness” to “alcohol problems”. The questionnaire measures, via three items each, “difference” (example “How different do you think a person with an alcohol problem is, compared to everyone else in the general population?”), disdain (example “How disrespected do you think a person with an alcohol problem is, compared to everyone else in the general population?”) and blame (example “How responsible do you think people with an alcohol problem are for their illness?”). The score on each item is summarized to a total score, where the minimum score is 9 and maximum 81, and a higher score indicates a higher level of stigma. The total score will be used in the analyses in the present study.

Attitudes towards others’ help-seeking for AUD were measured with the question: “What would you do if you were concerned that someone you knew had developed an alcohol problem?” The participants were given the following alternatives and answered yes or no to each: “I would not know what to say or do”, “Speak to them and support them, as well as I can”, “Help them to seek treatment”, “Seek possibilities for help on the Internet”, “Share my concerns with others in the social circle”, “I would avoid them”, and “I would not do anything and hope they or someone else solved the problem”. All alternatives were used in the analyses, but some were grouped. The groups of attitude items were made from theoretical decisions, where the authors discussed and reached consensus in which items that measured similar behavioral aspects of support. The groups were decided after data was collected, and before the statistical analyses were performed. The following two alternatives were grouped together in the analyses and named “Active strategy”: “Speak to them and support them, as well as I can” and “Help them to seek treatment”. The following two alternatives were grouped together in the analyses and named “Avoidance strategy”: “I would avoid them” and “I would not do anything and hope they or someone else solved the problem”. The five outcomes were: “I would not know what to say or do”; “Active strategy”; “Avoidance strategy”; “Seek possibilities for help on the Internet” and “Share my concerns with others in the social circle”. The grouped outcomes were rated “yes” if at least one of the included items were endorsed.

### Data analyses

After describing the sample as whole and separately per gender, using chi-square tests to assess association between categorical variables and t-test for the stigma total score, logistic regression models were used to investigate the association between the total score of stigma and the five outcomes, which measure attitudes towards help-seeking (“Active strategy”, “Avoidance strategy”, “I would not know what to say or do”, “Seek possibilities for help on the Internet”, “Share my concerns with others in the social circle”). The outcomes were dummy-coded. The data was inspected by visually plotting each exposure and the logit of outcome to check for linearity. When evidence of a non-linear relationship was found, restricted cubic spline transformation was used to model the relationship between the exposure, level of stigma, and each outcome [31]. Stigma was modeled, using Harrell’s recommendation [32], with three knots, spaced out over the 10th, 50th, and 90th percentile, corresponding to the following scores: 26, 41 and 52. For visualization, the reference value was placed at the stigma value 45, allowing for nine evenly spaced out points on the x axis, with the reference centered. Odds ratios (OR) in the plot were calculated with 95% confidence intervals (CI).

The analyses were performed in two steps. First, the crude associations between public stigma and the outcome measures were calculated, separated by sex. Next, all analyses were adjusted for age category, education, and experience of alcohol problems. P values for Bonferroni’s correction (0.05/20 = 0.0025) were calculated in order to decrease the risk of type I error. All analyses were carried out using Stata MP 16.1 (StataCorp LP, College Station, TX).

## Results

The demographic information for the participants can be seen in Table 1. The proportion of female and male participants was similar. Almost half of the participants were older than 50 years. One-third of the participants had children, and the majority of the participants had more than 12 years of education. 7% reported previously experienced alcohol problems themselves, the majority of them being male.

**Table 1:** Demographics of the whole sample and presented separated by sex

	Total	Female	Male	p-value
	N = 2895	N = 1535 (53.0%)	N = 1360 (47.0%)	
Age category				0.131
30–39 years	685 (23.7%)	379 (24.7%)	306 (22.5%)	
40–49 years	783 (27.1%)	426 (27.8%)	357 (26.3%)	
50–65 years	1427 (49.3%)	730 (47.6%)	697 (51.3%)	
Children				0.110
No	1858 (64.2%)	1006 (65.5%)	852 (62.7%)	
Yes	1037 (35.8%)	529 (34.5%)	508 (37.4%)	
Education				0.005*
Up to 12 years	358 (12.4%)	183 (11.9%)	175 (12.9%)	
Vocational training	763 (26.4%)	370 (24.1%)	393 (28.9%)	
> 12 years	1758 (60.7%)	973 (63.4%)	785 (57.7%)	
Missing	16 (0.6%)	9 (0.6%)	7 (0.5%)	
Previous alcohol problem (yes)	214 (7.4%)	69 (4.5%)	145 (10.7%)	0.000*
Missing	67 (2.3%)	23 (1.5%)	44 (3.2%)	
Stigma total score				
Mean	39.9 (10.3)	38.8 (10.3)	41.2 (10.2)	< 0.001*
25th percentile	33	32	34	
75th percentile	46	46	47	

**Fig. 1:**
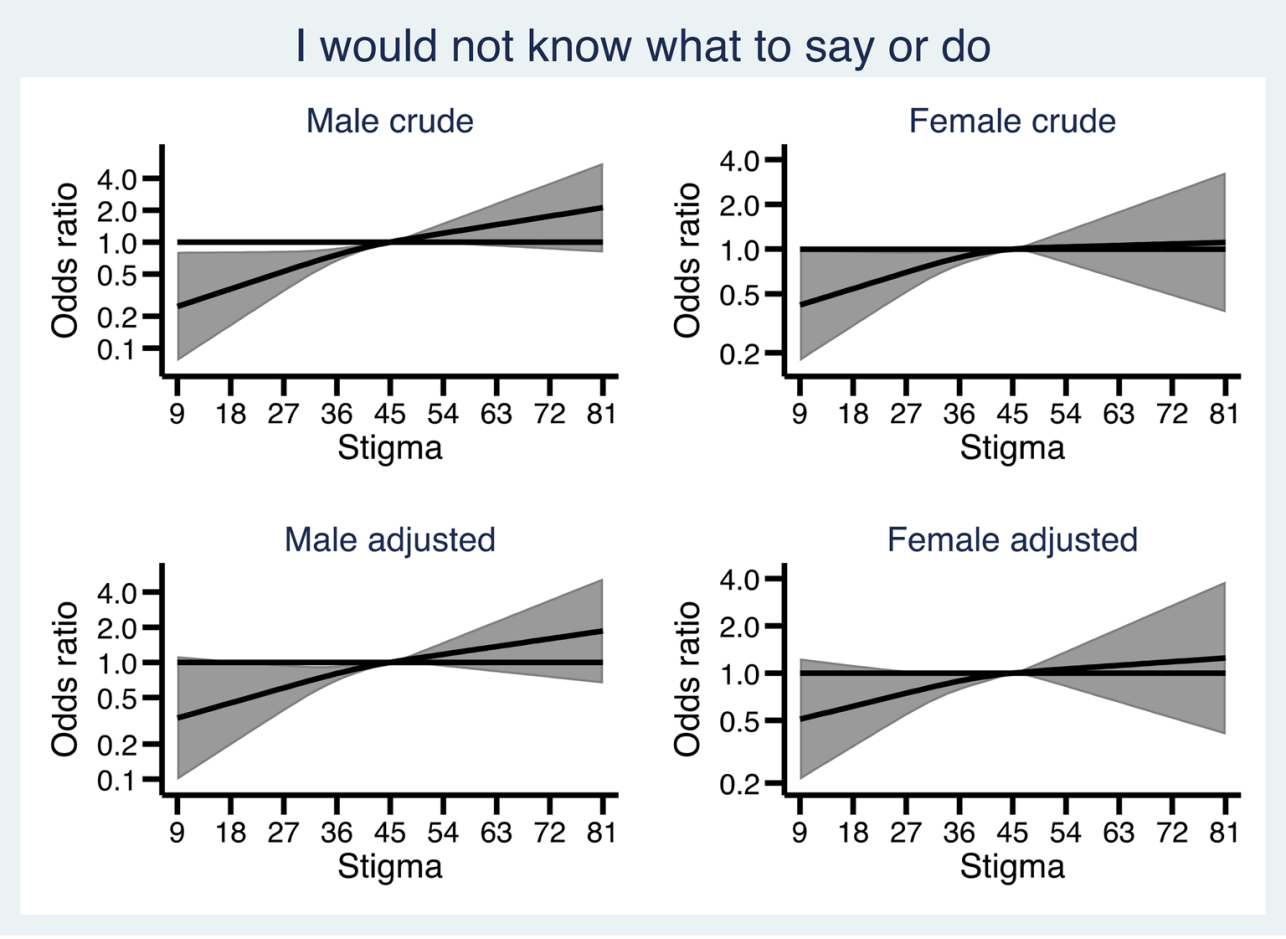
Association between level of public stigma and endorsing “I would not know what to say or do” presented separately for men and women. Adjusted for age category, education, and experience of alcohol problems

**Fig. 2:**
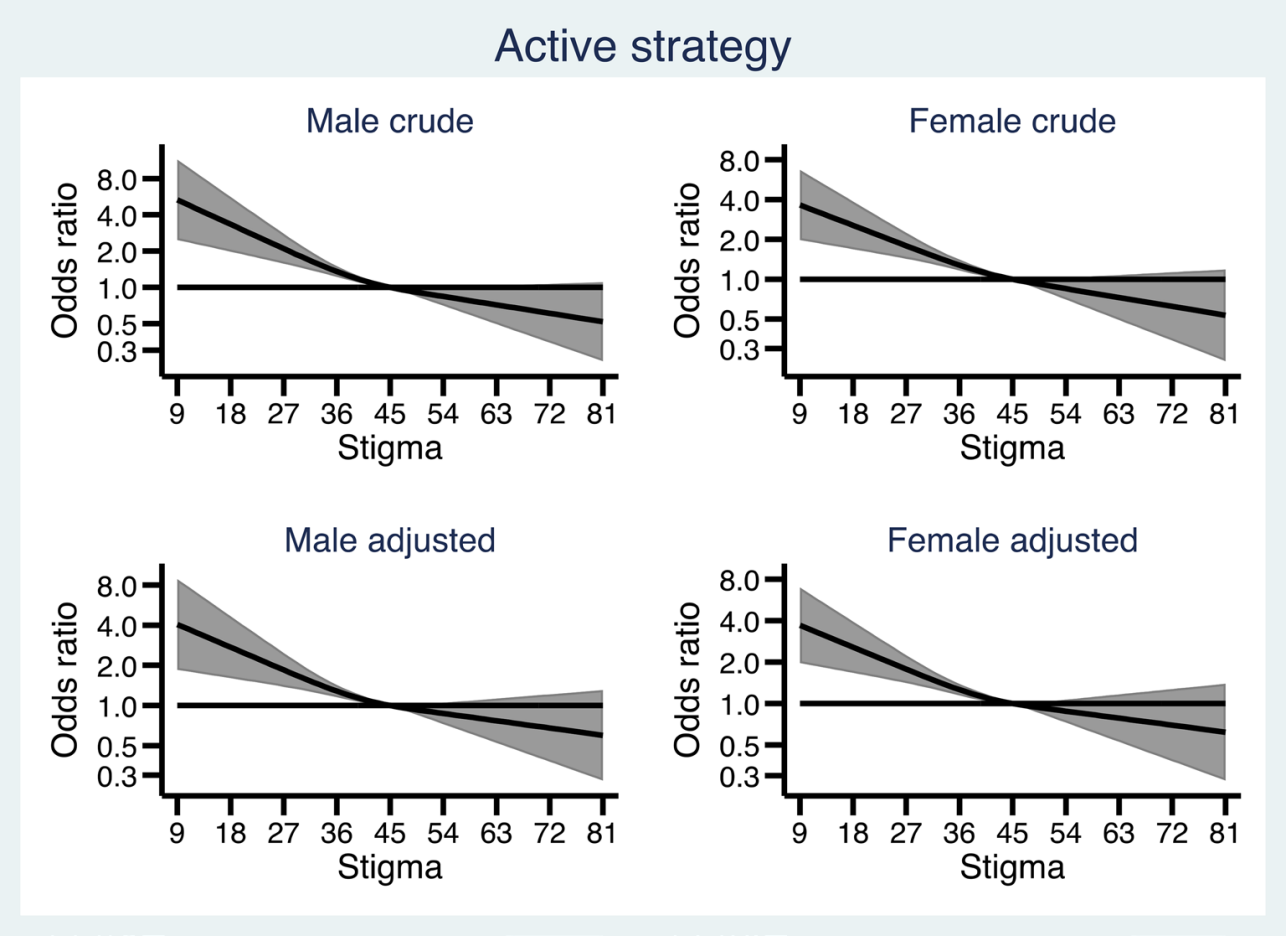
Association between level of public stigma and endorsing an “Active strategy” presented separately for men and women. Adjusted for age category, education, and experience of alcohol problems

**Fig. 3:**
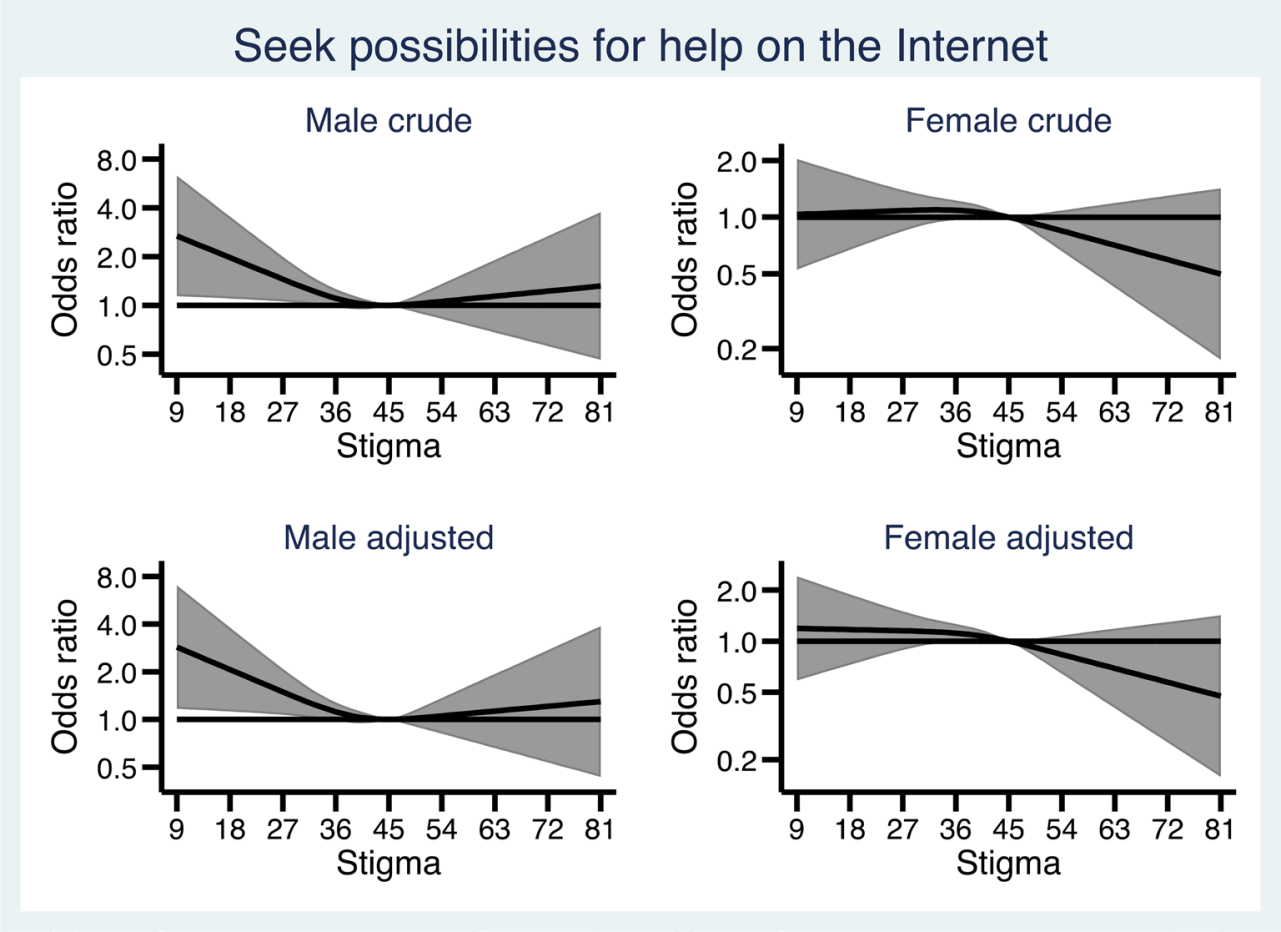
Association between level of public stigma and endorsing “Seek possibilities for help on the Internet” presented separately for men and women. Adjusted for age category, education, and experience of alcohol problems

**Fig. 4:**
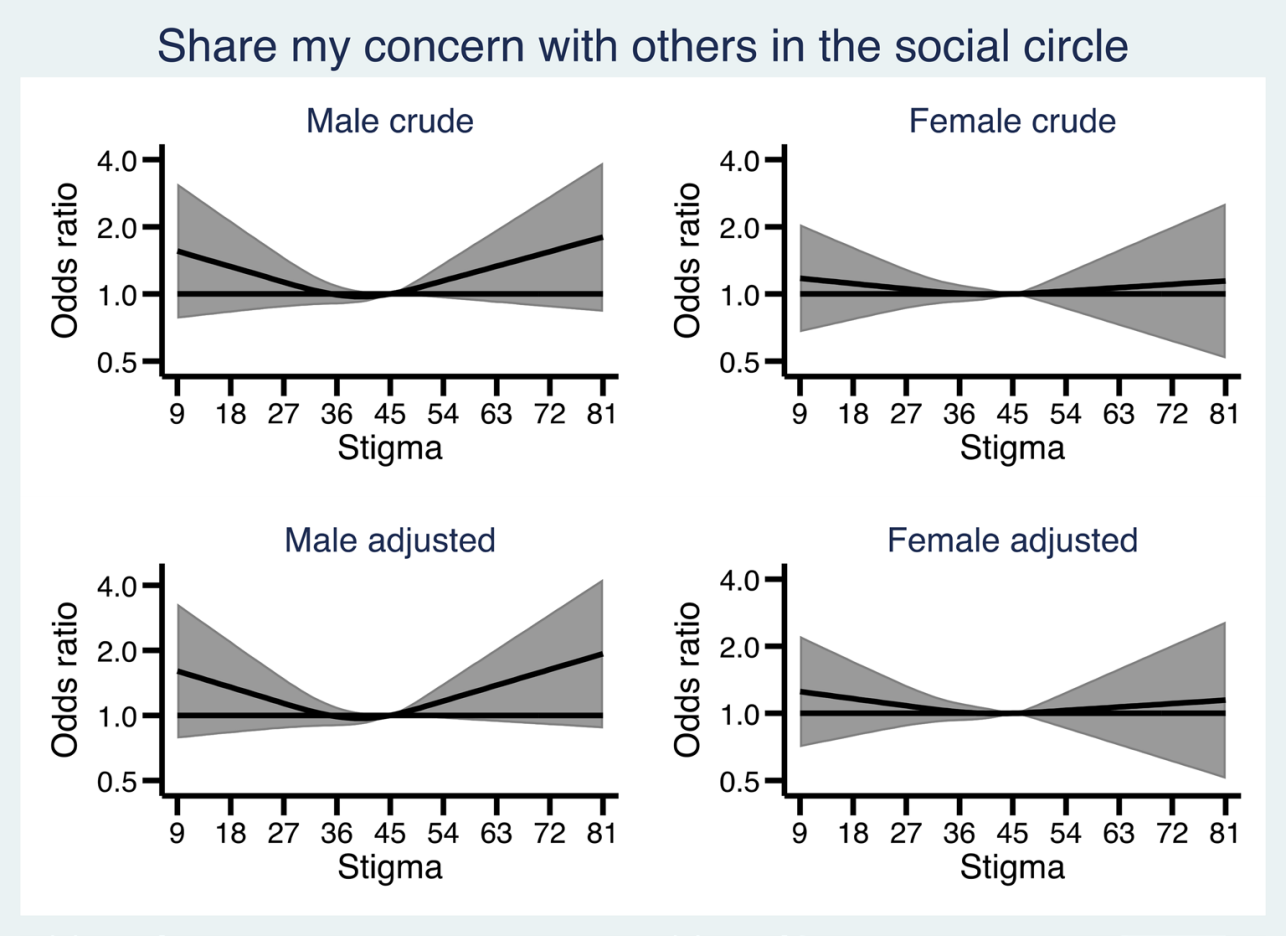
Associations between level of public stigma and endorsing “Share my concern with others in the social circle” presented separately for men and women. Adjusted for age category, education, and experience of alcohol problems

**Fig. 5:**
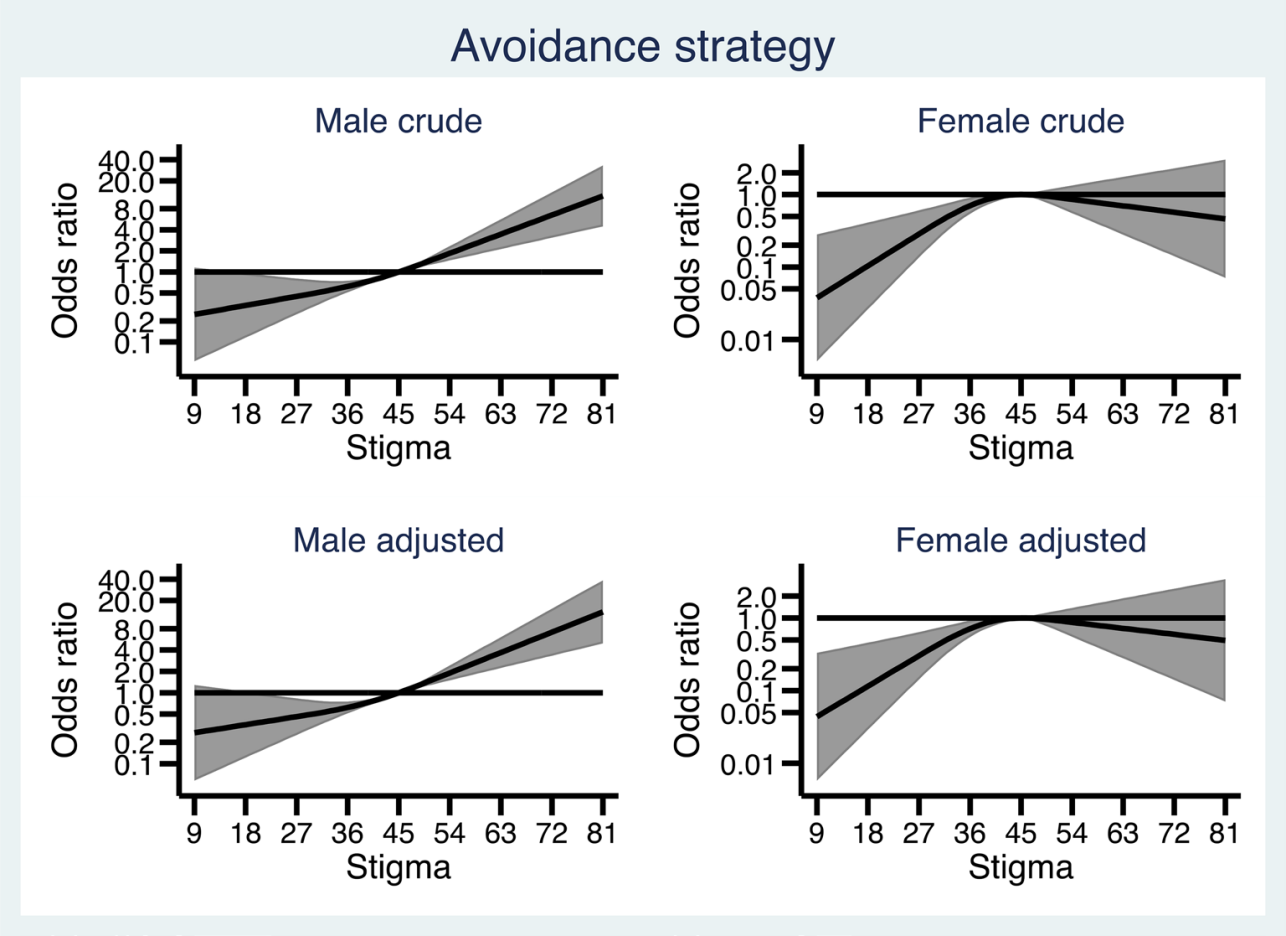
Associations between level of public stigma and endorsing an “Avoidance strategy” presented separately for men and women. Adjusted for age category, education, and experience of alcohol problems

Figures 1, 2, 3, 4 and 5 show how the male and female participants anticipated reacting if someone they know developed an alcohol problem, and how this was related to level of stigma, measured by the Difference, Disdain & Blame scale. In Additional file 1: Appendix S1, the estimates and p-values for the overall statistical model for each outcome are presented.

As can be seen in Fig. 1, in the crude model a lower level of stigma was associated with a lower probability of *not knowing what to say or do* among men. When adjusting for age, education, and personal experience of alcohol problems, there was no association, either for men or women, between perceived stigma and *not knowing what to say or do*.

However, as seen in Fig. 2, among both males and females, a lower stigma score was associated with a higher probability of an active strategy. Participants who endorsed the lowest stigma score had up to four times as high odds to endorse *an active strategy*.

Figure 3 shows that among men, lower stigma was associated with higher odds of *seeking possibilities for help on the Internet*. Among women, there were no associations between stigma and *seeking possibilities on the Internet*.

As seen in Fig. 4 , there were no associations between stigma and the probability of *sharing my concern with others in the social circle*.

Finally, Fig. 5 show that among men, higher stigma was associated with markedly higher odds of *an avoidance strategy*, and there was a trend towards lower stigma being associated with a lower probability of *an avoidance strategy*. Among women, lower stigma was associated with lower odds of endorsing *an avoidance strategy*.

## Discussion

The aim of this study was to investigate the associations between stigma and attitudes towards others’ help-seeking for AUD; a topic that to our knowledge has not been studied before. Moreover, the aim of our study was to explore possible differences associated with gender. Previous studies have examined the associations between stigma and own treatment-seeking; however, contextual factors such as attitudes in the general population towards others’ treatment-seeking have not previously been explored.

The results showed a rather clear association between stigma and attitudes towards others’ help-seeking for AUD. Lower level of stigma was associated with a higher probability of prosocial behaviors and an active strategy towards others’ help-seeking, including addressing and supporting the affected persons, and supporting them in seeking treatment. There were few gender differences: women, compared to men, seemed to a lesser degree to be impacted by level of stigma. Among men, a higher perception of stigma was associated with a markedly higher probability of *avoidance strategy*, and there was a trend towards lower perceived stigma associated with a lower probability of an *avoidance strategy*. Among women, lower perceived stigma was associated with a markedly lower probability of endorsing an *avoidance strategy*. Avoidance behaviors and the desire for social distancing from individuals with AUD can be seen both as a consequence of stigma and part of the stigma process [1].

Our findings highlight two important areas which are intertwined with one another: firstly, the importance of reducing the stigma associated with AUD, and secondly, social support for help-seeking for AUD. We will first discuss how stigma can be reduced.

### Reducing stigma

Prior research has shown that the stereotype of differentness decreases empathy and increases anger towards the group affected by stigma [29]. In turn, these processes lead to increasing disdain. It has been suggested that stigma can be reduced by targeting perspective-taking, i.e., increasing the understanding of how others perceive a situation. The hypothesis is that perspective-taking can increase the understanding of and empathy towards the stigmatized group, which in turn can decrease the level of stigma. Interventions that, on a general population level, have shown effectiveness in reducing stigma have had two different foci: one, to increase knowledge via education and two, to increase social contact with the stigmatized group [33, 34]. Both these foci can increase perspective-taking and empathy. The level of empathy towards a stigmatized person has also been found more predictive of prosocial behaviors when the helper and the stigmatized person belong to the same in-group [35, 36]. This is also in line with findings that the closer social network seems to be most important in reducing stigma, rather than statements from celebrities and media attention [37].

Another possible aspect to explore further is the messages around AUD and treatment-seeking. The emphasis on biological causes for AUD has been associated with a lower level of social distancing and blame, but also with higher perceived dangerousness [1]. In addition to investigating how messages around causes of AUD influence stigma, another path to explore further is how AUD is depicted. Traditionally, AUD has often been described in dichotomized categories—either someone fulfills the diagnostic criteria for the diagnosis, or not. This separation can reinforce the perception of differentness and increase stigma. With the change to DSM-5, severities of AUD was introduced, which implicates that fulfilling the diagnosis, i.e., having an alcohol problem, is a matter of continuum rather than a dichotomized separation [38]. To use continuum beliefs, rather than separated categories, for messages around AUD and treatment-seeking may be particularly relevant, as continuum beliefs has been found to reduce stigma for psychiatric disorders [39]. We suggest these aspects are further explored in future studies concerning stigma and support for others’ help-seeking.

### Social support for help-seeking

A well-known model of coping strategies among those surrounding individuals with AUD is the “stress-strain-coping-support (SSCS) model” [40]. It describes three coping styles: tolerant-inactive, including coping strategies as covering up for the individual with AUD; engaged coping, involving an active strategy towards the drinker with the aim of reducing the drinking, and withdrawal coping, which describes distancing oneself from the drinker [41]. Although the SSCS primarily is a family health model, it may be relevant to apply the model to our findings. Our findings suggest that lower stigma is associated with a higher probability of coping strategies based on engagement, while higher stigma is associated with coping strategies of distancing. Thus, since from a a public health perspective, active social support is considered to be of importance for the individual’s treatment-seeking, the question is how we, on a general population level, can lower stigma and improve positive attitudes and willingness towards actively supporting individuals suffering from AUD to seeking help and treatment.

### Limitations

This is a cross-sectional study, where it is not possible to draw any conclusions about causality or temporal direction of the associations between stigma and support for other’s help seeking. We suggest future studies address this limitation by using a longitudinal cohort design. Another limitation is the use of self-report measures, which do not always correlate with actual behaviours. Questions on stigma, alcohol problems and support for other’s help seeking, can all can be perceived as sensitive, and pose an increased risk of socially desirable answers. Due to social desirability, it is expected that level of stigma and experience of alcohol problems are underreported and that support for other’s help seeking is overreported, also in this type of anonymized survey. This increases the risk that associations between low level of stigma and higher level of support for other’s help seeking is attenuated. To reduce the risk of social desirability response bias, a stigma questionnaire emphasizing the measures of differentness was used, which is considered to impose less risk of biased answers [42, 43]. Previous studies show that individuals familiar with AUD and those with lived experience of AUD are less likely to endorse stigmatizing attitudes towards others with AUD [44]. A strength of this study is that a question about previous experiences of alcohol problems was included among the items and could, thus, be adjusted for in the analyses. However, only 7% of the participants self-reported experience of previous alcohol problems, which is markedly lower compared to national Danish estimates where 17% in the adult population endorse AUD [45]. The measure used in the current study was not validated, and the results raises concern of the validity of this measure. Moreover, recruitment of participants via a market research company could increase the risk for selection bias, where individuals with AUD possibly could be underrepresented.

Studies from other areas of research suggest that gender differences can be attenuated and sometimes stems from social desirability response bias, where women seem more sensitive for social desirability response bias compared to men [46]. This poses a risk also in our study, and we suggest future studies include measures of social desirability.

One limitation in the study is the high number of analyses. When accounting for Bonferroni´s correction the results suggesting that lower stigma was associated with lower odds of endorsing an avoidance strategy among women were not significant. However, no other results changed.

Perceptions of AUD and stigma are complex phenomena, where synergistic effects between these and different factors such as socioeconomic position, gender, age are expected. A strength of the current study is that these factors were included, together with a large sample size.

## Conclusion

There is a clear association between stigma and support towards others’ help-seeking for AUD in this general population sample. The results highlight the need to reduce the stigma associated with AUD, and promote engagement towards others’ treatment-seeking.

### Supplementary Information

**Additional file 1: Appendix S1.** Estimates for the overallstatistical model, presented for each outcome.

## Data Availability

The dataset generated and/or analysed during the current study are not publicly available due to ethical reasons but are available from the corresponding author on reasonable request.
