# Supplementary material for: The associations between public stigma and support for others’ help-seeking for alcohol use disorder: a cross sectional study in the general Danish population
Source: Addict Sci Clin Pract. 2023 Aug 4;18:46. doi: 10.1186/s13722-023-00400-2 (PMC10403820; doi:10.1186/s13722-023-00400-2)
Supplement: Supplementary file 1 — Additional file 1: Appendix S1. Estimates for the overallstatistical model, presented for each outcome. [file 13722_2023_400_MOESM1_ESM.docx]

Appendix S1

Estimates for the overall statistical model, presented for each outcome.

| I would not know what to say or do | | | | |
| --- | --- | --- | --- | --- |
|  | crude | *p* | adjusted | *p* |
| Male | chi2(2)=13.17 | 0.0014** | chi2(2)=7.74 | 0.0795 |
| Female | chi2(2)=5.07 | 0.0795 | chi2(2)=3.47 | 0.1764 |
|  | | | | |
| Active strategy | | | | |
|  | crude | *p* | adjusted | *p* |
| Male | chi2(2)=34.13 | 0.0000** | chi2(2)=21.77 | 0.0000** |
| Female | chi2(2)=30.18 | 0.0000** | chi2(2)=26.34 | 0.0000** |
|  | | | | |
| Seek possibilities for help on the Internet | | | | |
|  | crude | *p* | adjusted | *p* |
| Male | chi2(2)=5.31 | 0.0703 | chi2(2)=5.54 | 0.0627 |
| Female | chi2(2)=2.15 | 0.3415 | chi2(2)=3.00 | 0.2230 |
|  | | | | |
| Share my concern with others in the social circle | | | | |
|  | crude | *p* | adjusted | *p* |
| Male | chi2(2)=2.70 | 0.2589 | chi2(2)=3.10 | 0.2122 |
| Female | chi2(2)=0.34 | 0.8446 | chi2(2)=0.59 | 0.7444 |
|  | | | | |
| Avoidance strategy | | | | |
|  | crude | *p* | adjusted | *p* |
| Male | chi2(2)=42.75 | 0.0000** | chi2(2)=42.63 | 0.0000** |
| Female | chi2(2)= 10.76 | 0.0046* | chi2(2)=9.78 | 0.0075* |

*p value <0.05 and >0.0025

**p value <0.0025 (Bonferroni’s correction)
